# *Plasmodium falciparum* finds the winning combination

**DOI:** 10.1016/j.jbc.2021.100526

**Published:** 2021-04-01

**Authors:** Alvan C. Hengge

**Affiliations:** Department of Chemistry and Biochemistry, Utah State University, Logan, Utah, USA

**Keywords:** drug resistance mechanism, hybrid protein, DADE-ImmG, Plasmodium falciparum, malaria

## Abstract

After 3 years of laboratory drug pressure in the presence of a picomolar inhibitor, the parasite *Plasmodium falciparum* developed a combination strategy of gene amplification and mutation to regain viability. The mutation observed led to a dysfunctional enzyme, but new research reveals the clever mechanism behind its success. Not that we needed a reminder of nature’s creativity in the time of a pandemic.

There are more than a hundred species of *Plasmodium*, protozoan blood parasites that infect vertebrates. The parasite *Plasmodium falciparum* is transmitted to humans through the bite of a female *Anopheles* mosquito and causes the most dangerous form of malaria. The emergence of drug-resistant parasites has beleaguered efforts to eradicate malaria. The approach of combination drug therapy, a widely practiced countermeasure to deal with the emergence of resistant organisms, has been used against *P. falciparum* in an attempt to block resistance. However, the parasite is resistant to nearly all current therapies, including the currently WHO-recommended combination-based approach based on artemisinin (1).

*P. falciparum* parasites are purine auxotrophs and salvage purines *via* a pathway in which its purine nucleoside phosphorylase (*Pf*PNP) has an essential role in breaking down inosine scavenged from the host to form the purine precursor hypoxanthine. In 2004, an extensive study of the transition state of inosine cleavage by *Pf*PNP using kinetic isotope effects was carried out (2). Analysis of the data indicated *Pf*PNP catalysis goes through a highly dissociative, S_N_1-like transition state, which led to the design of a transition state analog, 4’-Deaza-1’- Aza-2’-Deoxy-1’-(9-Methylene)-Immucillin-G (DADMe-ImmG). The compound is a picomolar inhibitor of *Pf*PNP, and crystal structures of the enzyme–inhibitor complex (PDB ID: 3PHC) revealed how it binds more tightly than the substrate inosine (PDB ID: 2BSX) (3). Subsequently, it was shown that *P. falciparum* infections in a primate malaria model could be cleared by oral administration of DADMe-ImmG (4). It had been assumed that a transition state analog inhibitor would limit the development of resistance because mutations that disrupt the inhibitor function should also disrupt catalysis, but whether this holds true in the complicated biological milieu has not been established.

A previous study reported findings from resistance selection experiments after 3 years of continuous drug pressure (5). The results revealed the parasite’s version of combination-based “therapy”, synergistically allying targeted gene amplification with a point mutation presumed to provide functional resistance. Genomic sequencing of resistant organisms showed the presence of the M183L mutation in about one-half of the sequences read, the other half being WT *Pf*PNP copies. Unexpectedly, the purified enzyme containing the M183L mutation was severely compromised, with catalytic efficiency too low to support parasite survivability. So what was the advantage provided by the evolved mutant?

Minnow *et al.* (6) noted two additional aspects of *Pf*PNP that could factor in. First, *Pf*PNP functions as a hexamer, consisting of a trimer of dimers. Second, a crystal structure (5) indicated that Met183 resides between the catalytic site and the subunit interface, and the mutation allows the side chain of Tyr160 to enter the active site and hinder substrate binding. These observations led to the hypothesis that the resistant organism might utilize a hybrid form of *Pf*PNP with a combination of the mutant and native subunits (Fig. 1). The authors tested their proposal by engineering a fusion protein composed of native and M183L mutant subunits joined with a short peptide linker. The kinetic properties of the mutant/native fusion protein were characterized and compared with native *Pf*PNP, using a tethered version of the native enzyme as a control for effects of the peptide linkers.

**Figure 1:**
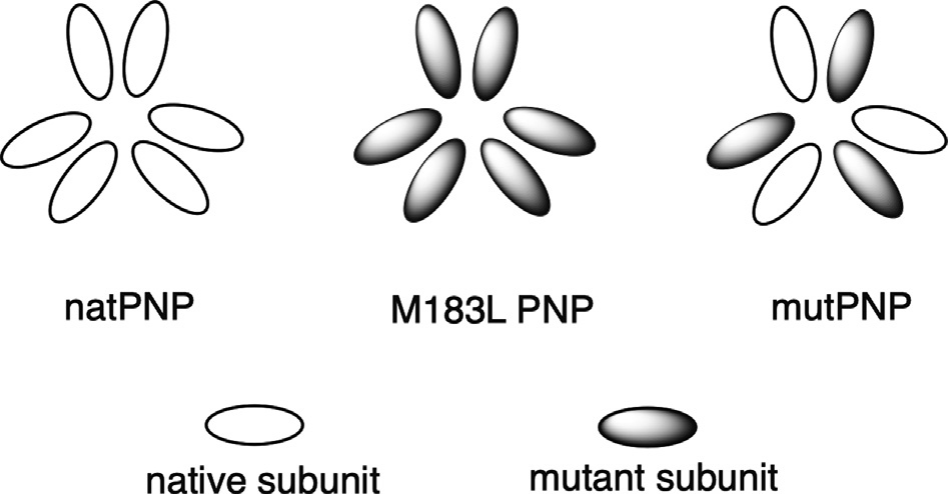
**Subunits in native and variant *Pf*PNP, which assemble in a trimer-of-dimer structure.** In native *Pf*PNP (natPNP) and M183L PNP, all subunits are either native or contain the M183L mutation. The resistant organism expresses equal amounts of native and M183L mutant subunits which can combine within single hexamers as depicted (mutPNP).

Although native *Pf*PNP has a high catalytic efficiency (*k*_cat_/*K*_M_) of 3.5 × 10^5^ M^−1^s^−1^, fully mutated M183L *Pf*PNP is functionally inert—its *K*_M_ value is increased, and the *k*_cat_ is decreased compared with the WT, resulting in a 17,000-fold decrease in catalytic efficiency. The binding of the DADMe-ImmG transition state analog is also weaker; its dissociation constant increases from 670 pM in native *Pf*PNP to 26 μM in the M183L variant. Thus, although the mutation weakens inhibition, the enzyme is also too weak a catalyst for survival of the organism. However, the catalytic efficiency of the fused hybrid composed of alternating native and M183L subunits is only 9-fold lower than the native fused enzyme, with a similar increase in its *K*_i_ for DADMe-ImmG, confirming the notion that affinity for a transition state analog should track inhibition of catalysis. The kinetics of inhibition are also altered in the hybrid: with native *Pf*PNP, the inhibitor induces a slow-onset isomerization process leading to the formation of a tightly bound enzyme–inhibitor complex while the hybrid enzyme exhibits a much shorter slow-onset phase. The authors also tested the effect of a slightly longer peptide linker and the insertion of a protease cleavage site to release the fusion and more accurately represent the cellular assembly. A PAGE gel analysis of the cleaved construct suggested it has the same hexameric structure as the native enzyme; removal of the linkers modestly increased the kinetics of both the native and mutant enzymes. These data, combined with a 12-fold gene amplification found in the resistant organisms, reveal a combination of countermeasures that restores the catalytic capacity for purine salvage to a level comparable with that in the WT parasite. Although this is not the first example of an organism developing resistance using a combination of strategies, the usage of mixed-subunit hybrid hexamers to productively implement an otherwise fatal mutation is unusual.

The authors point out that although the typical malaria treatment period lasts from days to weeks, it is far shorter than the 3 years needed for the resistance strategy to develop and conclude that the likelihood of such resistance developing in the field is very low. These findings remind us, however, of the relentlessness of microorganisms to evolve ways to survive under pressure. This combination of mutation, hybridization, and gene duplication to combat a drug was akin to picking a winning combination in the lottery. There are two ways to better one’s odds in a lottery: play it long enough or play it with more numbers. Although incubation times will be shorter in the field, the number of “incubators,” patients undergoing treatment, is large. These results have obvious implications for the present pandemic and warn us that humans do not own the natural world intellectual property rights for combination therapy.

## Conflict of interest

The authors declare that they have no conflicts of interest with the contents of this article.
